# EFFECT OF A HOME-BASED EXERCISE PROGRAM ON SUBSEQUENT FALLS IN OLDER ADULTS WITH COGNITIVE FRAILTY

**DOI:** 10.1093/geroni/igad104.1978

**Published:** 2023-12-21

**Authors:** Teresa Liu-Ambrose, Ryan Falck, Jennifer Davis, Karim Khan, Kenneth Madden

**Affiliations:** University of British Columbia, Vancouver, British Columbia, Canada; The University of British Columbia, Vancouver, British Columbia, Canada; University of British Columbia - Okanagan, Kelowna, British Columbia, Canada; University of British Columbia, Vancouver, British Columbia, Canada; University of British Columbia, Vancouver, British Columbia, Canada

## Abstract

Cognitive frailty is characterized by concurrent physical frailty and mild cognitive impairment. and increases the risk for falls. Whether exercise can reduce falls in older adults with cognitive frailty is unknown. We examined the effects of a home-based exercise intervention on subsequent falls among community-dwelling older adults with cognitive frailty and a history of falls. A secondary of a 12-month randomized controlled trial among older adults aged ≥70 years with a fall in the last 12 months. Participants were randomized to either 12 months of home-based exercise (EX; n=172) or usual care (UC; n=172). For this analysis, we only included participants who were classified as cognitively frail based on a Short Physical Performance Battery (SPPB) score ≤ 9/12 and a Montreal Cognitive Assessment score < 26/30. Our primary analysis examined the effect of EX on self-reported falls over 12 months. The secondary analysis examined whether higher exercise adherence, or dose, benefits physical frailty among the EX participants. At baseline, 192 participants were classified as cognitively frail (EX=93; UC=99). Falls rates were lower in EX participants vs. UC participants (IRR=0.65; p=0.042). At 12 months, in the EX group, SPPB score was significantly higher among participants with high adherence vs. those with low adherence (estimated mean difference: 1.22; p=0.004). Exercise is a promising strategy for reducing subsequent falls in older adults with cognitive frailty and a history of falls. Greater exercise adherence, or dose, may reduce physical frailty in this population at high risk for disability.

